# A Parent-Focused Pilot Intervention to Increase Parent Health Literacy and Healthy Lifestyle Choices for Young Children and Families

**DOI:** 10.5402/2013/619389

**Published:** 2013-05-12

**Authors:** Sasha Fleary, Robert W. Heffer, E. Lisako McKyer, Aaron Taylor

**Affiliations:** ^1^Department of Psychology, Texas A&M University, 4235 TAMU, College Station, TX 77845, USA; ^2^Department of Health and Kinesiology, Texas A&M University, 4222 TAMU, College Station, TX 77843, USA

## Abstract

Health literacy affects caregivers' ability to engage in preventive health care behaviors for themselves and their children. Studies suggest that health literacy among low-income families needs improvement, and this possibly contributes to disparities in preventive health care rates. Additionally, parents and caregivers may not be able to provide or seek preventive health care for their children because of lack of knowledge and skills to do so effectively. This study designed and piloted an intervention that delivered to parents of young children (1) health literacy information in an experiential manner and (2) practical skills to engage their families in healthy lifestyle choices. Specifically, the intervention focused on diet/nutrition, physical activity, sleep hygiene, parenting, and mental wellness. Postintervention improvements were noted for factual knowledge for diet/nutrition, physical activity, and sleep, beliefs about diet/nutrition, and the relationship between mental health and stress. Additionally, postintervention improvements were noted for general knowledge and beliefs about sleep, knowledge about the relationship between sleep and health, knowledge about common childhood sleep problems, and parents' bedtime interactions with children. The efficacy of the intervention should be evaluated on a larger, more diverse sample in the future with considerations for multiple health behavior change in the evaluation.

## 1. Introduction

Although some genetic variables may account for obesity, it is one of the most preventable diseases. Health literacy (HL) and preventive care are fundamental in preventing obesity. The Institute of Medicine [1] reported that racial and ethnic minorities, GED certificate recipients, nonnative English speakers, older adults, individuals with below 12th grade education, and individuals with low incomes are more likely to have low HL. Additionally, children whose families meet at least one of these criteria also tend to have low preventive health and be at increased risk for preventable diseases such as obesity and cardiovascular diseases. This study targeted the selective sample of parents with social factors which make them susceptible to having lower HL and making fewer healthy lifestyle choices for their children. The goal of this study was to develop and pilot an HL intervention for underserved families with young children by targeting barriers to preventive health such as education and financial resources and providing hands-on tools for promoting preventive health. 

Insufficient HL poses many barriers to the prevention of obesity among children since it may lead to improper diet/nutrition, inadequate physical activity (PA), and misidentification of weight status [2–5]. Conversely, higher parental HL and overall literacy is related to healthy diet/nutrition and lower prevalence of overweight children [5, 6]. Barriers to healthy diet/nutrition include lack of knowledge, training, and experience in preparing, buying, and introducing healthier foods to children, as well as inadequate support from family members [7]. Parental behaviors and attitudes toward diet/nutrition, weight status, sedentary activity (SA), PA, and parents' and children's eating habits are also major targetable areas related to obesity [5, 8–11].

Insufficient sleeping hours are also related to childhood obesity. Researchers have found a relationship between short sleep duration, decreased leptin levels, increased ghrelin levels, and increased hunger and appetite, suggesting that reduced sleep causes changes in these appetite regulatory hormones [12–14]. Chaput and colleagues [14] found that children who did not get sufficient sleep were 3.45 times more likely to be obese than other children. Sleep duration, therefore, may be considered a modifiable risk factor for obesity and a targetable area for prevention. In addition to obesity, sleep loss has significant effects on children's neurobehavioral functioning, academic performance, and school adjustment [15–19].

 In developing a parent-focused prevention intervention to increase healthy lifestyle choices for young children and families, it is imperative that mental wellness is addressed. Many researchers have found mental health problems in parents to be related to reduced preventive health care service-seeking and behaviors for preschool children [20, 21]. Maternal depression is also related to sleep problems in children [18, 22]. Additionally, Kavanaugh and colleagues [20] and Minkovitz and colleagues [21] found that mothers with depressive symptoms tended to have fewer years of education, lower incomes, and single-parent households and to be from ethnic minorities, all of which are risk factors for low HL and decreased preventive health.

Studies have shown that parenting styles regarding PA and diet/nutrition affect children's PA and healthful eating habits. Positive reinforcement, monitoring, and authoritative parenting are associated with healthful eating habits and increased PA, while controlling, permissive, and authoritarian parenting styles are associated with reduced PA, increased unhealthy eating, increased BMI scores, and weight status [11, 23–25]. Similarly, Johnson and McMahon [26] concluded that effective authoritative parenting may be necessary in interventions focused on reducing sleep problems in children, providing further evidence for incorporating parenting skills in the intervention. 

## 2. The Framework for the Intervention

This study was originally conceptualized using Bronfenbrenner and Morris [27] Bioecological Model which emphasizes the roles of systems in children's socioecological health. However, the recently developed Framework for Children's Health Promotion proposed by Mistry and colleagues [28] incorporates the Bioecological Model, has more predictive value, and directly emphasizes HL. The Framework for Children's Health Promotion links early childhood policies and programs to subsequent health outcomes. The framework considers the role of policies and programs (e.g., Head Start) in improving family (i.e., financial resources, time, psychological resources, and human capital which includes HL), and community (i.e., institutional resources, collective efficacy) capacities to build on the foundations of health. According to the Framework, the foundations of health include responsive caregiving, safe and secure environments, appropriate and adequate nutrition, and health-promoting behaviors, all of which influences biological mechanisms that shape health across the lifespan. In addition to systems that influence the child, the Framework considers social, economic, and cultural determinants of health (e.g., poverty, education, and discrimination). These determinants of health make our target population very susceptible to having low HL and health disparities. The goal of our intervention was to minimize the negative effects of the determinants of health and increase family capacities for the foundations of health. We expected that, by targeting family capacity, children's own preventive health behavior would be modified immediately and across the lifespan due to modeling and exposure in the home. To target family capacity, we provided tools and knowledge in the intervention to maximize families' existent time and financial resources. In addition to health knowledge, psychological resources and human capital were targeted via the positive parenting strategies parents learned to help them encourage preventive health and healthy lifestyle choices for their children. 

The intervention was innovative because of its experiential nature; we linked knowledge to behavior by creating an intervention where parents had the opportunity to instantly practice what they learned. Additionally, the experiential nature of the intervention reduced the negative effects of the determinants of health since the materials were developed to address the barriers to preventive health and healthy lifestyle choices for the targeted population. In doing so, health knowledge was disseminated in a manner that was resilient to our targeted population's barriers, and this goes beyond the interventions already in place to promote healthy lifestyle choices. By incorporating the literature with community input on risk and protective factors in the intervention development, we aimed to utilize the community's strengths to further promote healthy lifestyle choices. We hypothesized that preventive health behavior would be predictive of HL. We also hypothesized that there would be increases in HL and preventive health behaviors for diet, PA, sleep, and mental wellness postintervention and at one-month followup.

## 3. Method

### 3.1. Participants

Twenty-one mothers (*M*_age_ = 23.14, SD = 5.45) of young children were recruited from local Head Start, Early Head Start, and teenage pregnancy agencies via flyers distributed by the agencies' staff and word of mouth for participation in the intervention. Thirteen mothers (62%) completed the intervention, and 9 (69%) completed measures at 1-month followup. The retention rate from the first session to followup was 43%. Participants were of varied ethnicities (~29% Caucasian, ~29% African-American, and ~33% Hispanic). Seventy-one percent of participants had at least a high school education, 20% earned $20 000 or less per year, 52% were unemployed, 76% identified English as their primary language, and 62% were Women, Infants, and Children (WIC) voucher recipients. 

### 3.2. Measures

Demographic variables measured included socioeconomic and sociocultural information about the family. Standardized measures of HL measure reading ability and are poor measures of HL [1, 29]. For this reason, we developed a study-specific measure of HL that included factual statements about the targeted health behaviors. All subscales, except for Knowledge (which was scored correct [1] or incorrect [0] and summed), were rated on a 4-point Likert scale ranging from Strongly Disagree to Strongly Agree; responses were reverse-coded where necessary (higher scores = more knowledge), and average scores were calculated and are presented in Table 1. Brief effectiveness measures were also developed for each targeted behavior to evaluate the extent to which participants practiced the skills after they left the session. All study-related materials and measures created by the authors were at or below a 7th grade reading level. 

#### 3.2.1. Diet/Nutrition HL

Knowledge (7 items) tested participants' knowledge about the food groups, serving sizes, and good and bad fats. Disease (5 items) assessed participants' knowledge about the relationship between diet/nutrition and diseases. General Diet assessed participants' knowledge about the purpose and importance of diet/nutrition (4 items). Beliefs (31 items) was an overall average score derived from participants' beliefs about general statements about diet/nutrition, food, eating, General Diet, and Disease. 

#### 3.2.2. PA HL

 Knowledge (5 items) tested participants' knowledge about the amount of time adults and children should spend engaging in PA, SA, and bone- and muscle-strengthening exercises. 

Disease (3 items) assessed participants' knowledge about the relationship between diseases and PA. Beliefs (20 items) was an overall average score derived from participants' beliefs about general statements about the relationship between PA and children's wellbeing, engaging in PA, the importance and purpose of PA, and Disease.

#### 3.2.3. Diet Nutrition and PA Behaviors

 Items were taken from Sallis' Active Where? Parent Child Survey and Amherst Health and Activity Study Survey to measure participants' behaviors. Both measures have demonstrated adequate internal consistency, test-retest reliability, and construct and discriminant validity [30–33]. Family influence questions asked participants to report how much they engaged in the behavior in the last week on a 5-point interval (0 = Never, 1 = 1-2 days, 2 = 3-4 days, 3 = 5-6 days, and 4 = 7 days). Family influence on fruits and vegetables (*FI F&V; *4 items) and low-fat foods (*FI LF; *4 items) examined how often participants influenced their children's fruits, vegetables, and low-fat food consumption by providing them with these foods and encouraging them to eat them. Family influence on PA (*FI PA;* 4 items) and SA (*FI SA*) examined participants' influence on their children's activity by providing them with opportunities and encouraging them to participate in PA and reducing opportunities and discouraging them from participating in SA, respectively. Diet/Nutrition PA Lifestyle (DNPA Lifestyle; 10 items) examined participants' diet/nutrition and PA lifestyle choices for the home and were rated on a 4-point Likert scale. 

#### 3.2.4. Sleep Hygiene

Knowledge (2 items) tested participants' knowledge on how much sleep children and adults should get per night. Disease (4 items) assessed participants' knowledge and beliefs about the relationship between sleep and health. Child Sleep Problems (6 items) assessed knowledge and beliefs about common sleep problems in children. Beliefs (17 items) was an overall average score derived from participants' beliefs on the relationship between sleep and children's wellbeing, Disease, and Child Sleep Problems. 

The Parental Interaction Bedtime Behavior Scale [34] (PIBBS) is a 17-item measure of parents' techniques at children's bedtime. Parents rate their behavior on a 5-point frequency scale ranging from Never to Very Often. The measure yields five scales and a total score. Active Physical Comforting* (PIBBS-A)* is a measure of parents' strategy of actively putting their child to sleep. Encourage Autonomy* (PIBBS-B)* is a measure of parents' strategy of having the child put himself to sleep. Settle by Movement* (PIBBS-C)* is a measure of parents' strategy of using movement to settle the child. Passive Physical Comforting* (PIBBS-D)* is a measure of parents' strategy of being physically present for the child to go to sleep without engaging in active comforting. Social Comforting* (PIBBS-E)* is a measure of parents' strategy of using verbal and/or social interaction to put the child to sleep. The scale has reasonable reliability (reliability alpha = 0.71) and discriminant and construct validity [34]. 

#### 3.2.5. Parenting and Mental Wellness

*Stress* (5 items) assessed participants' knowledge about the relationship between stress, mental wellness, and caring for children. Parent-Child Relationship (3 items) assessed participants' knowledge about the effects of parent-child relationships. Diseaseassessed participants' knowledge about the relationship between unhealthy habits and children's wellbeing (4 items). 

Parent depressive symptoms and stress were measured using the Center for Epidemiological Studies-Depression Scale [35] (CES-D) and Perceived Social Stress-14 [36] (PSS-14), respectively. The CES-D is a 20-item measure of depressive symptoms over the last week on a 4-point scale ranging from Rarely or None of the Time (<1 day) to Most or All the Time (5–7 days). The CES-D has proven to be reliable with good internal consistency, acceptable test-retest reliability, good concurrent, and construct validity and is reliable across ethnic groups. The PSS-14 is a 14-item measure of individuals' perception that the situations in their lives are stressful. Individuals are asked to report on their thoughts and feelings in the last month on a 5-point scale ranging from Never (1) to Very Often (5). The PSS-14 has good internal consistency (reliability alpha = 0.75) and construct validity [36]. 

Children's behavioral functioning was assessed using the eyberg child behavior inventory [37] (ECBI). The ECBI is a 36-item parent rating of child behavior problems on a 7-point scale ranging from Never to Always. The ECBI yields an Intensity Scale (frequency of problem) and a Problem Scale (tolerance of behavior and distress behavior causes). The Intensity Scale was used for this study. 

Children's HRQoL was assessed using the 15-item Pediatric Quality of Life Inventory-Short Form-Parent Report for Toddlers [38, 39] (PedsQL). Parents are asked to report their children's physical, social, emotional, and school functioning on a 5-point scale ranging from Never to Almost Always. The measure has shown good reliability (reliability coefficient = 0.9) and validity. The physical and emotional subscales were used for this study. 

### 3.3. Procedures/Research Design

The project was approved by the Texas A&M University Institutional Review Board (IRB) and all participants completed informed consents prior to participation. In keeping with the tenets of community-based participatory research approaches (i.e., meaningful community involvement in the early phases of planning), three community-based focus groups (Head Start facilitators, English-speaking, and Spanish-speaking Head Start parents) were conducted to gather information about parents' risk and protective factors for HL and adopting healthy lifestyle choices. Focus groups were transcribed and content analyzed using thematic analyses. The information gathered was compared with what was already outlined in the literature, and any themes not addressed in the literature were added to the list of ideas to be incorporated in the intervention. 

Intervention development was iterative and highly dependent on parents' identified risk and protective factors as well as stated needs. The intervention aimed to improve HL and healthy lifestyle choices and practices for five targeted areas: diet/nutrition, PA, sleep hygiene, parenting, and mental wellness. These areas were targeted because they were identified by focus group participants as areas of high need. Head Start administrators reviewed the initial intervention manual and materials and provided feedback about readability and content. Changes were made based on this feedback, and the intervention was conducted with five parents who initially participated in the focus groups. These parents provided feedback about content, readability, and practicality for carrying out the skills in the home. This feedback was incorporated in the intervention, and the intervention was finalized (see Table 2 for sample of intervention details).

The intervention consisted of five weekly sessions. Groups were offered in familiar community locations on mornings and evenings and lasted approximately 90 minutes. Daycare and meals were provided. All participants completed a demographic questionnaire and pretest measures at the beginning of the intervention. Participants completed posttest measures at the end of the intervention and one month after the completion of the intervention. Measures took approximately 30–45 minutes to complete. In addition to intervention materials, participants received monetary compensation weekly for their time ($15–$20).

### 3.4. Statistical Analyses

Descriptive statistics were computed. Multiple regression analyses were used to evaluate whether parents' preventive health behaviors for themselves and their children were related to HL. Multilevel model analyses were conducted to test for postintervention improvements in HL and preventive health behaviors for parents and children. All analyses were conducted using SPSS 16.0 [40].

## 4. Results

### 4.1. Diet/Nutrition

 Of the diet/nutrition HL variables, Knowledge and Disease were predicted by diet/nutrition behaviors (Table 3). Specifically, FI F&V was negatively related to Knowledge. Conversely, WIC enrollment and education were positively related to Knowledge. FI F&V was somewhat positively related to Disease. WIC enrollment was negatively related to Beliefs and General Diet. 

Regarding hypothesis 2, participants' knowledge of the relationship between diet/nutrition and disease (Disease) was significantly lower at posttest (*B* = −0.32, *t* = −2.07, and *P* = 0.05) when compared to pretest. Participants' overall beliefs about diet/nutrition (Beliefs); did not change from pretest to posttest or from posttest to followup; however, it improved from pretest to followup (*B* = 0.23, *t* = 2.35, and *P* = 0.03). Regarding participants' knowledge about the importance and purpose of diet/nutrition (General Diet), an improvement was seen at posttest (*B* = 0.53, *t* = 3.27, and *P* < 0.01) and followup (*B* = 0.56, *t* = 3.01, and *P* < 0.01), while no significant change was noted between posttest and followup. 

No significant behavioral changes were found. However, regarding practicing the skills taught, 78% used serving sizes, 94% served more “Go” foods, and 83% made substitutions to make meals healthier during the week after the diet/nutrition module was conducted. 

Additionally, 89% modeled healthy eating, 89% allowed children to help prepare meals, 61% planned meals, and 100% encouraged their children to eat healthier in the week following diet/nutrition module. 

### 4.2. Physical Activity

 Of PA HL variables, only Knowledge was negatively related to FI SA (Table 3). No other relationships between knowledge and behavior were found. 

Regarding postintervention changes, participants' factual knowledge about PA (Knowledge) improved from pretest to posttest (*B* = 0.85, *t* = 2.50, and *P* = 0.02); however, no significant changes were found between pretest and followup and from posttest to followup. No other significant changes were found. However, participants reported engaging in vigorous PA (58%), moderate PA (66%), using chore charts (33%), and games booklet (50%) to encourage PA during the week after the PA module was conducted. Additionally, 75% limited their children's television watching, and 80% of those who limited television replaced television watching with PA. Overall, 82% of parents reported encouraging children to engage in PA during the week following the PA module. 

### 4.3. Sleep Hygiene

 Regarding behavior predicting HL (Table 3), education was positively related to Beliefs, Disease, and Child Sleep Problems. In addition to education, settling by movement (PIBBS-C) and active physical comforting (PIBBS-A) were negatively and positively related to overall beliefs about sleep (Beliefs), respectively. Similarly for Disease, active physical comforting (PIBBS-A) was positively related to HL whereas the relationship was somewhat negative for settling by movement (PIBBS-C). Regarding Child Sleep Problems, income, and active physical comforting (PIBBS-A) they were positively related to HL while the relationship was negative for passive physical comforting (PIBBS-D) and encouraging autonomy (PIBBS-B). 

Regarding postintervention changes, participants' overall beliefs about sleep (Beliefs; pretest to posttest: *B* = 0.26, *t* = 2.48, and *P* = 0.02) pretest to followup: (*B* = 0.27, *t* = 2.25, and *P* = 0.03), factual knowledge about sleep (Knowledge; pretest to posttest: *B* = 0.57, *t* = 3.34, and *P* < 0.01), knowledge of the relationship between sleep and health (Disease; pretest to posttest: *B* = 0.47, *t* = 2.74, and *P* = 0.01), and child sleep problems (Child Sleep Problems; pretest to posttest: *B* = 0.44, *t* = 3.69, and *P* ≤ 0.001; pretest to followup: (*B* = 0.46, *t* = 3.33, and *P* < 0.01)) improved. Education was also positively related to Beliefs (*B* = 0.11, *t* = 2.67, and *P* = 0.02) and Disease (*B* = 0.14, *t* = 2.66, and *P* = 0.03). 

Regarding participants' bedtime behavior change, bedtime behaviors were lower at followup than at pretest (*PIBBS-A*: *B* = −28.33, *t* = −3.80, and *P* ≤ 0.001; *PIBBS-B*: *B* = −26.36, *t* = −3.23, and *P* < 0.01; *PIBBS-C*: *B* = −29.32, *t* = −3.15, and *P* < 0.01; *PIBBS-D*: *B* = −24.09, *t* = −3.13, and *P* < 0.01; *PIBBS-E*: *B* = −34.57, *t* = −3.18, and *P* < 0.01) and posttest (*PIBBS-A*: *B* = −23.03, *t* = −2.96, and *P* < 0.01; *PIBBS-B*: *B* = −37.22, *t* = −4.26, and *P* < 0.01; *PIBBS-C*: *B* = −40.19, *t* = −4.07, and *P* < 0.01; *PIBBS-E*: *B* = −34.08, *t* = −2.93, and *P* < 0.01). However, no significant changes were found from pretest to posttest. 

Regarding sleep practice following module, 67% of participants reported following a sleep routine, and the routine was followed for between three and seven days. Participants reported bedtime problems in their children (62%) and reported using positive strategies to handle bedtime problems during the week after the sleep module.

### 4.4. Parenting and Mental Wellness

 Stress and parent-child relationship were predicted by participants' mental wellness and children's behavior and HRQoL (Table 3). Specifically, for the HL of stress, the relationship was positive for education, perceived stress, and child physical HRQoL but negative for depression, child behavior problems, and child emotional HRQoL. Regarding HL for parent-child relationships, the relationship was positive for education, perceived stress, and child physical HRQoL but negative for income, depression, child behavior problems, and child emotional HRQoL. 

Regarding postintervention changes, participants' knowledge about the relationship between stress and mental health (*Stress*) increased from pretest to posttest (*B* = 0.28, *t* = 2.33, and *P* = 0.03) although no significant change was found from posttest to followup. Children's emotional HRQoL improved from pretest to followup (*B* = 10.58, *t* = 2.30, and *P* = 0.03) and from posttest to followup (*B* = 15.98, *t* = 3.33, and *P* < 0.01). Participants' perceived stress was lower at followup than at posttest (*B* = −3.64, *t* = −2.56, and *P* = 0.02). Education was related to *Stress *HL (*B* = 0.16, *t* = 2.61, and *P* = 0.02), Disease (*B* = 0.11, *t* = 1.83, and *P* = 0.09), and perceived stress (*B* = −1.66, *t* = −2.28, and *P* = 0.04). 

Regarding positive behavior modification strategies after parenting module, 70% introduced a new behavior, 55% eliminated an unhealthy behavior, 27% maintained a healthy behavior, 27% decreased an undesirable behavior, and 100% worked on improving their relationship with their children in the week following the parenting module.

## 5. Discussion

### 5.1. Diet/Nutrition

As stated previously, there was reason to believe that behaviors would predict HL for diet/nutrition. The results were partially supportive of this hypothesis. We included WIC as a covariate because participants enrolled in WIC programs are offered at least two voluntary nutrition education classes at time of certification [41]. Although the use and the effectiveness of these classes are debatable [42], we anticipated that it would affect participants' HL. As expected, being enrolled in WIC was positively related to factual knowledge; however, it was negatively related to Beliefs and General Diet. These findings suggest that WIC enrollment should be considered when carrying out and evaluating interventions involving diet and nutrition of families. WIC programs should also be considered as a target for HL interventions. Education only being predictive of Knowledgeand income not being a significant predictor of HL is contrary to what has been presented in the literature [1]; we propose that our findings may be due to the homogeneity of the sample and the small sample size making it difficult to achieve significant results. Improvements in HL scores from pretest to followup suggest that the intervention may be helpful in improving HL for diet/nutrition at least one month after intervention. Participants' reports of the skills they practiced after the diet/nutrition module provide evidence of the effectiveness of the diet/nutrition module of the intervention to translate knowledge into behavior. However, we are unable to comment on how long these skills were practiced after intervention. This limitation will be addressed when the intervention is carried out on a large scale via weekly measurements of practical behaviors.

### 5.2. Physical Activity

 We proposed that there would be a strong relationship between PA behaviors and HL. Education and income were not significant correlates of HL. Contrary to what was expected, parents with more factual knowledge about PA did not discourage their children from engaging in SA. Although unexpected, these findings allude to research showing that there is no direct relationship with PA and SA [43]. The intervention will be modified to actively address SA knowledge and behavior before it is disseminated on a larger group.

 Regarding the efficacy of the intervention, preliminary findings suggest that the intervention may have been successful in immediately improving factual knowledge about PA. However, the intervention should be modified to achieve more long-term HL improvements. PA behavior results suggest that the intervention was not successful at improving behavior as measured by standardized measures. However, similar to diet/nutrition, participants' reports on PA-related behaviors in the week following the PA module provide evidence of the effectiveness of the PA intervention. 

### 5.3. Sleep Hygiene

Results of sleep behaviors explaining HL for sleep are consistent with other findings on the relationship between HL and health outcomes [44, 45]. Participants' strategy of settling their children by movement was negatively related to general knowledge and beliefs about sleep and the relationship between sleep and health providing confirmation of the relationship between HL and ability to engage in preventive health behaviors [46]. Noteworthy is the negative relationship between participants' knowledge about common sleep problems in children and their strategy of engaging in passive physical comforting. Some common sleep problems such as bedtime problems and night waking are maintained by parent behaviors such as parents' presence in the room [47]; thus, it is no surprise that participants who lack knowledge about these problems engage in behaviors that encourage continuation of these problems. Also noteworthy is the active physical comforting being positively indicative of Beliefs, Disease, and Childhood Sleep Problems. This relationship was not expected. However, one possible explanation for these results might be that participants may view this behavior as an acceptable bedtime behavior due to problematic cognitions (e.g., difficulty setting limits, doubt about parenting competence) about child sleep or as a response to child temperament [26, 48]. Consistent with HL research [1], income and education were positively related to participants' knowledge about common sleep problems confirming that these variables are risk factors for HL for sleep. 

Regarding the efficacy of the intervention, HL for sleep improved from pretest to posttest and from pretest to followup, but no significant improvements was seen from posttest to followup. These results provide evidence that the intervention may be useful on a wider scale in improving sleep HL. Factual knowledge about sleep improved from pretest to posttest but decreased from posttest to followup suggesting that although the intervention may have successfully improved factual knowledge immediately, the improvement was short term. 

Parent interaction behaviors at bedtime decreased from pretest to followup and from posttest to followup suggesting that the intervention may have successfully reduced participants' bedtime interactions with children that are indicative of sleep problems [34]. Noteworthy is that this pattern was seen for participants' strategy of encouraging autonomy and was not a goal of this intervention. We predicted that this behavior will increase over time as seen in Morrell and Cortina-Borja [34], and because it sets children up for healthy bedtime behaviors, this pattern may be indicative that participants do not have to engage in any bedtime behaviors with their children over time (which might be due to the intervention). Although these results question the extent to which the intervention was successful at increasing positive bedtime behaviors, participants' reports on their handling of bedtime problems during the week following the sleep module provide some support of real-world applications of the skills learned in the intervention.

### 5.4. Parenting and Mental Wellness

Child behavior and mental wellness being indicative of HL were partially confirmed. Consistent with the general HL literature [1], education was positively related to participants' knowledge about stress/mental health and parent/child relationships. Participants with more knowledge about stress/mental health and parent/child relationships reported fewer depression symptoms and fewer child behavior problems. Knowledgeable participants also tended not to be depressed, and research has shown that depression is related to parents seeking preventive health services for their children [20, 21]. These findings reiterate the need to address the psychological wellbeing of parents when trying to improve their HL as well as capacity for making positive decisions for their children. 

Regarding the efficacy of the intervention, the intervention had very limited success in changing behavioral functioning and stress variables. Knowledge about the relationship between stress and mental health improved from pretest to posttest suggesting that the intervention may have been successful in improving knowledge immediately but the intervention should be modified to produce more long-lasting changes. Children's emotional HRQoL improved from pretest to followup and from posttest to followup suggesting some more lasting (1 month) effects of the intervention and may also be a reflection of 100% of participants reporting working on improving their relationship with their children. 

### 5.5. Limitations and Future Directions

 This study was a pilot of an intervention that will be carried out on a larger scale using a diverse sample in the near future. The large-scale study should include a comparison group since the lack of a comparison group is a limitation to interpreting the results of this pilot study. Additionally, the small homogeneous sample does not allow for comparisons among key sociodemographic variables. Therefore, the extent to which barriers such as education and income were addressed in the intervention cannot be fully determined. The ability to replicate these findings in nonhelp-seeking parents is also debatable. Evaluation of this intervention should include recruitment of a larger, representative sample and adopt a multisite approach without restricting the intervention to parents enrolled in Head Start or other agencies. The small sample size also meant that the study had very low power and significant relationships between variables, and postintervention improvements may have went undetected. The results of this pilot study will be used to calculate the sample size needed to achieve power, and this should be used as a guide for recruitment of a sample in further evaluations of this intervention. Future evaluations of the intervention would also include longer postintervention followup, and evaluation of the results in the context of multiple health behavior change due to the interrelatedness of the variables. 

The HL scales were validated on a college sample and consistently yielded Cronbach alphas above 0.60; however, Cronbach alphas in this parent sample ranged from 0.30 to 0.85. Future research will include validation of the scales on a larger parent sample before evaluating the intervention on a larger scale. Regarding measurement of behaviors, future research will include measures that are more sensitive to the behavior change targeted in the study, for example, using the 1-week-practice measure for evaluation of behavior change and skills usage throughout the assessment of the intervention.

## 6. Conclusions

The main objective of this study was to design and pilot an experiential intervention targeting HL and healthy lifestyle choices of parents of young children. Topics for the intervention were elicited from parents and Head Start facilitators and included diet/nutrition, physical activity, sleep, parenting, and mental wellness. The targeted behaviors varied in the extent to which improvements were made with the least postintervention change being seen for mental health and the most change being noted for sleep. However, 1-week-practice reports indicated that participants were practicing skills learned in session for all targeted behaviors. The efficacy of the intervention cannot be fully commented on due to the lack of a comparison group and limited power; however, the results provide evidence that the intervention may be valuable to underserved parents and should be evaluated further.

## Figures and Tables

**Table 1:** Descriptive statistics for all measures.

Variable	*α*	Pretest (N = 21)		Posttest (N = 13)		Followup (N = 9)	
		*M*	SD	*M*	SD	*M*	SD
Diet/Nutrition							
HL food knowledge	—	3.52	1.50	3.92	1.19	3.13	1.55
HL disease	0.40	3.35	0.38	3.11	0.59	3.25	0.55
HL diet Beliefs	0.81	2.90	0.35	3.23	0.37	3.28	0.43
HL general diet	0.54	2.61	0.59	2.99	0.58	3.31	0.57
FI fruits and vegetables	0.85	2.56	1.06	2.73	0.89	2.50	0.50
FI low fat	0.86	1.90	1.19	2.13	1.00	2.47	0.74
DNPA lifestyle	0.90	2.95	0.70	2.90	0.61	3.05	0.59
Physical activity							
HL knowledge	—	1.42	0.93	2.08	0.95	1.78	1.09
HL PA disease	0.30	3.08	0.44	3.18	0.57	3.33	0.53
HL PA beliefs	0.77	3.06	0.32	3.1.	0.32	3.30	0.40
FI PA	0.92	2.25	1.18	2.10	1.03	1.78	0.82
FI SA	0.71	1.79	0.96	2.17	0.72	1.98	0.48
Sleep							
HL knowledge	—	0.48	0.51	1.08	0.64	0.50	0.53
HL disease	0.54	2.70	0.53	3.27	0.55	3.17	0.56
HL beliefs	0.80	2.86	0.36	3.18	0.41	3.21	0.51
HL child sleep problems	0.57	2.61	0.44	3.13	0.37	3.15	0.55
PIBBS-A	0.78	41.44	26.18	34.85	19.83	14.81	21.15
PIBBS-B	0.21	44.17	21.30	54.17	25.00	16.67	23.94
PIBBS-C	0.31	34.38	27.77	46.88	34.18	8.33	17.68
PIBBS-D	0.10	40.00	27.08	29.17	24.62	18.06	19.87
PIBBS-E	0.85	60.00	28.85	61.46	32.07	29.17	32.33
Stress/mental wellness							
HL Stress	0.80	3.02	0.57	3.40	0.45	3.25	0.60
HL Relationship	0.72	3.30	0.50	3.31	0.55	3.33	0.56
HL Disease	0.85	3.16	0.51	3.19	0.53	3.03	0.87
CESD	0.89	33.33	10.04	31.15	9.29	33.50	8.49
PSS	0.57	39.75	6.30	39.00	5.19	36.22	8.47
PedsQL physical	0.89	72.89	31.55	79.58	24.54	85.56	26.15
PedsQL emotional	0.78	78.29	18.79	70.83	20.87	88.89	11.17
Eyberg ECBI	0.86	89.60	18.47	87.09	26.71	89.89	50.24

Note. HL: health literacy; PA: physical activity; SA: sedentary activity; FI: family influence; DNPA: diet/nutrition physical activity; PIBBS: parent interactive bedtime behavior scale; CESD: center for epidemiological studies-depression scale; PSS: perceived social stress-14; PedsQL: pediatric quality of life inventory; ECBI: Eyberg Child Behavior Inventory.

**Table 2:** Sample outline of intervention modules.

Week	Module	Sample objectives	Experiential examples	Sample materials*
1	Diet/Nutrition	(1) Understand what diet/nutrition is and its importance (2) Understand the link between diet/nutrition, school performance, diseases, and general health (3) Understand portions and daily food group needs and have the skills and tools to practice this (4) Time and finances will no longer be viewed as major barriers to healthy eating	(1) Measure portions and compare portions of fruits and vegetables and snacks (e.g., chips) that children are served (2) Positive parenting strategies for encouraging children to eat healthy foods (3) Meal planning and grocery shopping tips and practice (4) Label reading practice	(1) Scales, measuring cups, and spoons (2) Blank grocery shopping list with traffic light system and food groups incorporated (3) Pocket nutrition label reading guide (4) Cookbooks with healthy common recipes

2	Physical activity	(1) Understand what PA and SA are and PA importance (2) Understand the link between PA, school performance, diseases, and general health (3) Understand PA needs and SA limits and have the skills and tools to increase PA and reduce SA for themselves and their children	(1) Demonstrate the difference between light, moderate, and vigorous PA (2) Positive parenting strategies for encouraging PA and reducing SA	(1) Indoor physical activity game book (2) Sedentary activity monitor sheet (3) Common home items that can be used as exercise equipment

3	Sleep	(1) Understand the importance of sleep (2) Understand and identify sleep problems (3) Understand the link between sleep, school performance, diseases, and general health	(1) Develop sleep schedules (2) Positive parenting strategies for sleep problems	(1) Sleep schedules (2) Guide for handling sleep problems

4	Parenting	(1) Understand parent/child relationships and child temperament (2) Understand the link between child behavior, school performance, and general health (3) Know how to bring out the best in children	(1) Positive parenting strategies and tools for handling behavior problems and unhealthy habits (2) Practice handling behavior problems based on parent Q and A	Worksheets for handling behavioral problems with examples

5	Stress and mental wellness	(1) Understand the importance of mental wellness and its role in ability to care for children (2) Able to recognize psychological distress in children (3) Navigate the health care system	(1) Create manageable schedules (2) Stress reduction strategies (3) How to be prepared for children's doctor's appointments and get all questions answered	(1) CDs with progressive muscle relaxation, deep breathing, and imagery exercises (2) List of common well child visit questions for doctors (3) 52 week scheduling notepad

Note. PA: physical activity; SA: sedentary activity; *where possible materials were in color, contained more pictures than words, and laminated.

**Table 3:** Hypothesis 1: Multiple regression estimates for predicting health literacy from behavior.

	Knowledge	Disease	Beliefs	General diet	Sleep problems	Stress	Relationship
	*β*	*β*	*β*	*β*	*β*	*β*	*β*
Diet							
Education	0.58*	−0.16	0.17	−0.05			
WIC	0.57*	−0.23	−0.64**	−0.55*			
Income	**−**0.31	0.31	0.11	0.26			
FI F&V	−0.86*	0.74^†^	−0.14	0.20			
FI Low Fat	0.39	−0.15	−0.09	−0.58			
DNPA Lifestyle	0.20	−0.01	0.43	0.27			
* R* ^2^	*0.55 *	*0.51 *	*0.54 *	*0.41 *			
* F*	*2.62 * ^†^	*2.28 *	*2.57 * ^†^	*1.36 *			
PA							
Education	−0.28	0.22	0.22				
Income	0.09	−0.16	−0.02				
FI PA	−0.28	−0.31	−0.21				
FI SA	−0.54*	−0.23	−0.26				
DNPA lifestyle	−0.01	0.34	0.30				
* R* ^2^	*0.41 *	*0.27 *	*0.25 *				
* F*	*1.92 *	*0.94 *	*0.85 *				
Sleep							
Education	−0.14	0.71**	0.71**		0.56**		
Income	−0.25	−0.20	0.17		0.50**		
PIBBS-A	−0.57	1.45**	1.49**		1.28***		
PIBBS-B	−0.12	−0.05	−0.18		−0.44*		
PIBBS-C	0.15	−0.69^†^	−0.91*		−0.18		
PIBBS-D	−0.14	0.00	−0.06		−0.72**		
PIBBS-E	0.73	−0.35	−0.09		0.18		
* R* ^2^	*0.26 *	*0.77 *	*0.80 *		*0.89 *		
* F*	*0.46 *	4.29 *	5.17 *		10.40 **		
Behavior							
Education		0.52				1.00**	0.71***
Income		−0.46				−0.39^†^	−0.16*
PSS		0.97				1.52**	2.43***
CESD		−0.97				−1.54**	−2.20***
ECBI		−0.63				−0.58*	−1.06***
PedsQL physical		0.69				1.17**	1.45***
PedsQL emotion		−0.50				−0.93*	−1.55***
* R* ^2^		*0.29 *				*0.84 *	*0.99 *
* F*		*−0.33 *				*0.71 *	*0.98 *

Note. WIC: Women, Infants, and Children Program, FI: family influence, F&V: fruits and vegetables; PA: physical activity; SA: sedentary activity; DNPA: diet/nutrition physical activity; PIBBS: parent interactive bedtime behaviour scale; PSS: perceived social stress; CES-D: Center for Epidemiological Studies-Depression Scale; PedsQL: pediatric quality of life. ^†^ *P* < 0.10; *P < 0.05; **P ≤ 0.01; ***P ≤ 0.001.
